# Association of Gene Variants in Matrix Metalloproteinases and Their Tissue Inhibitors with Intraventricular Haemorrhage in Preterm Infants

**DOI:** 10.3390/ijms27062596

**Published:** 2026-03-12

**Authors:** Dawid Szpecht, Karolina Żyto, Gabriela Ciszek, Karolina Duczmal, Zofia Kowal, Kornelia Kręciszewska, Zuzanna Słowińska, Grażyna Kurzawińska, Anna Sowińska, Agnieszka Seremak-Mrozikiewicz

**Affiliations:** 1 Department of Neonatology, Poznan University of Medical Sciences, ul. Polna 33, 60-535 Poznan, Poland; 2 Faculty of Medicine, Poznan University of Medical Sciences, 61-701 Poznan, Poland; 86777@student.ump.edu.pl (K.Ż.); 87044@student.ump.edu.pl (G.C.); 86971@student.ump.edu.pl (K.D.); 87029@student.ump.edu.pl (Z.K.); 87023@student.ump.edu.pl (K.K.); 86847@student.ump.edu.pl (Z.S.); 3 Laboratory of Molecular Biology, Department of Perinatology, Poznan University of Medical Sciences, Polna 33, 60-535 Poznan, Poland; gkurzawinska@ump.edu.pl (G.K.); asm@data.pl (A.S.-M.); 4 Department of Computer Science and Statistics, Poznan University of Medical Sciences, 61-701 Poznan, Poland; ania@ump.edu.pl

**Keywords:** prematurity, intraventricular haemorrhage (IVH), matrix metalloproteinases (MMPs), tissue inhibitors of metalloproteinases (TIMPs), preterm birth, genetic factors, prematurity complications, gene variants, preterm infants

## Abstract

The objective of the present study is to examine the association between the presence of various forms of matrix metalloproteinase genes (*MMP-1*, *MMP-9*, *TIMP-1* and *TIMP-2*) and their tissue inhibitors, and the incidence of intraventricular haemorrhage (IVH) in premature neonates. The data for this study were obtained from samples of peripheral venous blood, which were collected and stored post-delivery. The techniques employed for the purpose of genotyping were polymerase chain reaction (PCR) and restriction fragment length polymorphism (RFLP). The population that was examined comprised 100 patients with a gestational age (GA) ranging from 22 to 33 weeks and birth weight (BW) ranging from 432 to 2100 g. In the cohort of enrolled patients, 48 cases of IVH were observed. As indicated by the findings of this study, the majority of observed correlations between *MMP-1*, *MMP-9*, *TIMP-1*, and *TIMP-2* variants and IVH did not demonstrate statistical significance, with the exception of the T allele of *TIMP1 rs4898*. Nevertheless, the findings of this study indicated a potential impact of these variants on the incidence of IVH. The present study suggests that further research is required to elucidate the role of *MMP*/*TIMP* polymorphisms in the aforementioned disease.

## 1. Introduction

Intraventricular haemorrhage (IVH) is one of the most common complications of preterm birth. The condition is characterised by the occurrence of bleeding within the germinal matrix, as defined by the first author. The germinal matrix is a brain region that is characterised by its high cellularity and vascularisation. During gestation, it is located beneath the lower vault of the lateral ventricles [1,2]. This region has been observed to regress during the process of brain development [3]. IVH is a common occurrence among premature infants. However, it is also rarely diagnosed among full-term newborns, usually due to the presence of the persistent germinal matrix or vascular malformations [1]. The incidence of this pathology has been shown to be inversely proportional to gestational age (GA) [4,5,6]. The potential for the identification of multiple risk factors associated with IVH in a group of preterm infants is a notable observation. It is an irrefutable fact that low birth weight (BW) and low GA are the underlying causes of many complications of prematurity, including IVH [7,8,9]. In the extant literature, a preponderance of authors draw attention to the genetic underpinnings of this condition. To date, the influence of polymorphism in the gene encoding vitamin K metabolism, as well as transportation, endothelial nitric oxide synthase and fibronectin 1, has been explored [9]. A number of studies have previously investigated the association between single nucleotide polymorphisms in the vitamin D receptor gene and IVH. However, these studies did not identify a significant relationship [10]. Furthermore, the impact of mutant genes implicated in inflammation has the potential to be substantial [11]. In the present study, the influence of genetic variability in the matrix metalloproteinase encoding genes is investigated. Metalloproteinases (MMPs) are zinc and calcium-dependent enzymes. A total of 23 MMP proteins are encoded by 24 distinct genes. A categorisation of these enzymes could be proposed, distinguishing between three collagenases, two gelatinases, three stromelysins, matrilysin, macrophage elastase and four membrane-type MMPs. MMPs are secreted as inactive proenzymes and are inhibited by tissue inhibitors of metalloproteinases (TIMPs). The function of these cells is subject to regulation by cytokines, growth factors and extracellular matrix (ECM) components. The function of matrix MMPs is the degradation of ECM proteins. This process is of crucial importance during the developmental phases, the growth stages, the phases of uterine cycling and angiogenesis. Degradation of ECM proteins can induce inflammation because of their chemotactic abilities [12,13]. As demonstrated by the study that analysed the activity of MMPs during hypoxic–ischemic brain damage in the immature rat, increased MMP activity was associated with damage to the blood–brain barrier (BBB) [14]. A breakdown of the BBB can result in increased penetration of toxic substances into brain cells and a heightened risk of bleeding in this area. It is imperative to emphasise that MMPs play a pivotal role in the degradation of collagen [13], a process that has been shown to stabilise blood vessels [15]. This finding suggests the potential involvement of MMPs in the pathogenesis of IVH. The present study employs the same patient cohort and methodological framework described by Choręziak-Michalak et al. (2023) [16]; however, while the previous work investigated retinopathy of prematurity (ROP), this analysis focuses specifically on IVH.

## 2. Results

### 2.1. Clinical Data

Demographic and clinical features of patients enrolled in the study trial are presented in Table 1. The study population consisted of 100 patients (47 female, 53 male) with median GA of 28 weeks (range 22–33 weeks) and median BW of 1080 g (range 432–2100 g). IVH was diagnosed in 48 patients (22 female, 26 male), including one patient with I grade IVH, 21 patients with II grade IVH, 22 patients with III grade IVH and four patients with IV grade IVH.

As shown in Table 1, the relationship between development of IVH and GA, BW, Apgar score, presence of birth asphyxia, infections (intrauterine, early, and late-onset), ROP, bronchopulmonary dysplasia (BPD) and mechanical ventilation therapy was observed. Grade of IVH correlated with GA, BW, the 5 min Apgar score, the use of mechanical ventilation and presence of BPD and ROP.

The reported *p*-values were adjusted for multiple testing correction using the False Discovery Rate (FDR) method (Supplementary Tables S1–S6). For a detailed comparison of nominal and adjusted values, please refer to the Supplementary Materials, which aligns with the significant results highlighted in the main text and tables.

### 2.2. Association Studies

Table 2 presents association between preterm infants affected by IVH as well as those without this complication and frequencies of studied gene variants. Only one variant proved to be statistically significant. No deviation from the Hardy–Weinberg equilibrium (HWE) was detected in both groups. The *TIMP1 rs4898T* allele occurred at a higher frequency in IVH cases in comparison to no-IVH cases (66% vs. 50%; OR 0.524 95% CI: 0.296–0.926; *p* = 0.026). Due to this allele’s linkage to the X chromosome, we conducted separate tests depending on sex, which showed the correlation for male newborns. The *TIMP1 rs4898T* allele occurred significantly more often in male neonates with IVH than in those without the condition (69% vs. 48%), suggesting a potential association (OR 0.413; 95% CI: 0.186–0.914; *p* = 0.029). No other statistically significant differences were observed. The *MMP9 rs17576G* allele was slightly less frequent in the IVH group relative to the no-IVH group. On the other hand, *TIMP2 rs2277698T* appeared more common in neonates with IVH in contrast to the other group. The significant results were put in bold whilst the other observations are presented in Table 2.

The association between the occurrence of IVH and studied variants was analysed with the use of logistic regression. The findings are illustrated in Table 3. Calculations included crude OR and adjusted OR (AOR) for BW, GA, mechanical ventilation, ROP, APGAR 5′, BPD. The analysis was performed for males and females separately in the *TIMP1* gene, as it is linked to the X chromosome. No statistically significant variations were found. A trend was found in male neonates for CC homozygotes in the *TIMP1 rs4898* gene (crude *p* = 0.06; OR 0.260; 95% CI: 0.064–1.056), although this association further lost significance after adjustment (AOR 0.305; 95% CI: 0.060–1.556; *p* = 0.153). The current results provide a foundation for future testing on a larger scale to confirm these preliminary observations. Other non-significant trends were observed for the *MMP9 rs17576* gene in the whole group (result borderline; AOR 0.348; 95% CI: 0.121–1.000; *p* = 0.05). The discussed results are presented in Table 3.

For Table 2 and Table 3, correction for multiple testing using the FDR approach (Holm–Bonferroni) was applied to the tested genetic models. After adjustment for multiple comparisons, none of the associations remained statistically significant. The initially significant associations observed for the *TIMP1 rs4898* variant (*p* = 0.026) in male newborns were no longer significant (FDR-adjusted *p* = 0.208). Similarly, a borderline association noted for the *MMP9 rs17576* variant (*p* = 0.05) after FDR correction did not remain significant (FDR-adjusted *p* = 0.250).

Table 4 presents a comparison between patients with I + II grade IVH and III + IV grade IVH and frequencies of *MMPs* and *TIMPs* alleles and genotypes. No deviation from the HWE in the genotype distribution was detected. Table 5 shows the association between grade of IVH and studied variants, analysed using logistic regression. Crude OR and AOR for BW, GA, 5 min Apgar score, use of mechanical ventilation and presence of ROP and BPD were calculated. The codominant model was chosen as the main model. Additionally, analyses were performed under dominant, recessive and overdominant models. No association between studied variants and the grade of IVH was found.

## 3. Discussion

Prematurity and its associated complication, IVH, represent significant challenges for global medicine [17]. IVH is characterised by the occurrence of bleeding within the germinal matrix [1,17]. Whilst it is generally accepted that the first and second grades of IVH rarely induce further complications and that neurodevelopmental prognosis is excellent, there is a growing body of evidence to suggest that the third and fourth grades may be more prone to inducing significant damage, including but not limited to obstructive, non-obstructive and post-haemorrhagic hydrocephalus, developmental impairment, cerebral palsy and seizures [1,5]. The issue of prematurity is a global concern, with 15 million infants being born each year [18]. On a global scale, the prevalence of IVH ranges from 5 to 52% among newborns delivered at or beyond the 28th week of gestation. However, the precise incidence of the condition is subject to variation depending on the continent and, within those continents, specific countries [17].

The objective of this study was to evaluate the association between variants of *MMP-1*, *MMP-9*, *TIMP-1* and *TIMP-2* genes and IVH in the population of Polish neonates whose functional role had previously been investigated. The *MMP-1 rs1799750* variant is characterised by an insertion or deletion within the promoter region. Research has indicated that the 2G/2G genotype is associated with elevated transcriptional activity of the *MMP-1* gene [19]. *MMP-9 rs17576* and *rs17577* are non-synonymous single-nucleotide polymorphisms resulting in amino acid substitutions Gln279Arg and Arg668Gln, respectively. The *rs17576* polymorphism leads to a Gln → Arg substitution within the fibronectin type II domain of MMP-9, a region that plays a critical role in substrate recognition and binding to extracellular matrix components such as fibronectin. Functional characterisation of MMP-9 polymorphisms has indicated that this amino acid substitution may alter interactions with ECM substrates and modulate enzymatic activity, supporting a functional relevance of *rs17576* rather than a purely marker effect [20]. In addition, previous studies have demonstrated that the fibronectin type II domains of gelatinases are essential for efficient binding and degradation of ECM proteins, underscoring the biological plausibility that sequence variation within this region may influence MMP-9-mediated ECM remodelling [1,21]. In contrast, the *rs17577* variant is located in the hemopexin domain, a region that has been implicated in modulating both substrate and inhibitor binding [20]. The *TIMP1 rs4898* variant is a missense mutation in the coding sequence that has been shown to influence TIMP-1 expression and circulating levels, potentially modifying MMP inhibition. Genetic interaction analyses with *MMP-9* haplotypes further suggest that *rs4898* may modulate ECM remodelling pathways [22]. The *TIMP2 rs2277698* polymorphism is a synonymous mutation, characterised by a C > T substitution at position 303 (Ser101). While the precise effects of this variant on gene expression remain to be elucidated, it has been hypothesised that it may influence splicing processes and modify transcriptional regulation [23]. The *TIMP2 rs55743137* variant is an intron variant resulting in a G > T substitution [24].

A thorough analysis of clinical data pertaining to IVH risk factors was conducted, revealing substantial disparities between preterm infants with IVH and the control group (free of IVH) across several pivotal parameters. Neonates with IVH were characterised by a lower GA (median 27 weeks vs. 29 weeks) and lower BW (median 955 g vs. 1243 g) compared to preterm infants without IVH. The findings of this study indicate that prematurity and low BW are statistically significant risk factors for the development of IVH [24]. Furthermore, lower Apgar scores in the first and fifth minute after birth (4 vs. 6 and 7 vs. 8, respectively), a higher incidence of perinatal asphyxia (16.7% vs. 3.8%), and increased use of mechanical ventilation (68.8% vs. 32.7%) in infants with IVH, compared to those without, underscore the impact of hypoxia and intensive respiratory support on the risk of IVH [25]. It is noteworthy that previous studies have likewise identified a significant association between low Apgar scores and the incidence of other complications of prematurity [16,26]. Additionally, a higher prevalence of intrauterine and late-onset infections was observed among neonates with IVH, thereby underscoring the role of infectious and inflammatory processes in the pathogenesis of IVH. Moreover, infants with IVH who were born preterm exhibited a greater frequency of complications such as ROP (68.8% vs. 11.5%) and BPD (56.3% vs. 21.2%). Further analysis of IVH severity demonstrated a correlation between more severe cases (particularly grades III and IV) and lower GA, lower BW, reduced Apgar score in the fifth minute after birth, increased utilisation of mechanical ventilation, and a higher prevalence of BPD and ROP. These variables were statistically associated both with the occurrence and greater severity of IVH in the analysed cohort.

Our primary analysis reveals a significant association between the T allele in the *TIMP1 rs4898* gene and the occurrence of IVH, with the mutation being substantially more frequent in affected neonates than in the control group. The variation was found to be statistically significant, with frequencies of 66% and 50%, respectively, corresponding to OR of 0.524 (CI: 0.296–0.926; *p* = 0.026). A similar association was identified in male infants with IVH, where the *TIMP1 rs4898T* allele was observed to be significantly more prevalent than in the male group without IVH (OR 0.413; 95% CI: 0.186–0.914; *p* = 0.029; 69% vs. 48%). No statistically significant association was observed among male CC homozygotes (*p* = 0.06; OR 0.26; 95% CI: 0.064–1.056). Moreover, this association did not remain statistically significant after multivariate adjustment (AOR 0.31; 95% CI: 0.060–1.556; *p* = 0.153), and therefore these results should be interpreted with caution.

In the present study, no statistically significant associations were observed between *MMP9 rs17576* and *TIMP2 rs2277698* polymorphisms and the occurrence of IVH. Similarly, no statistically significant association was found between *MMP1 rs1799750* or *MMP9 rs17576* variants and IVH grade. These findings do not support a relationship between these polymorphisms and IVH in the studied cohort.

MMPs are a family of zinc-dependent, structurally related proteolytic endopeptidases that mediate both physiological and pathological tissue remodelling. Their enzymatic activity is tightly regulated by specific TIMPs. In humans, over 20 MMP family members have been identified and characterised, each endowed with the capacity to degrade a broad spectrum of ECM proteins, thereby contributing to diverse processes such as embryogenesis, implantation, wound healing, inflammation, tumour progression, and angiogenesis, while simultaneously modulating bioactive molecules including cell-surface receptors, apoptotic ligands, and cytokines [27]. Beyond these well-established roles, the involvement of MMPs in neural tissue organisation and cerebral vascular development is increasingly recognised. Dysregulation of MMP activity has been hypothesised to heighten the structural vulnerability of the neonatal brain, potentially contributing to the pathogenesis of IVH. The endogenous regulators of this process, TIMPs, comprise four members—TIMP1, TIMP2, TIMP3, and TIMP4—which are 21–28 kDa proteins either secreted in soluble form or anchored to the ECM [28]. TIMPs reversibly inhibit MMP activity through their N-terminal domain, which folds intramolecularly to bind the MMP active site. In plasma, MMP activity can additionally be modulated by α2-macroglobulin. The MMP/TIMP system is critical for the maintenance of a delicate balance in ECM remodelling, vascular integrity and neural development, all of which are particularly critical in the context of neonatal brain vulnerability [29].

The extant literature does not provide unequivocal evidence for a direct association between *MMP-1* and *MMP-9* gene polymorphisms and the risk of IVH in neonates. However, there are relevant findings suggesting that MMP-1 may indirectly influence the development of IVH. For instance, research by Fujimoto et al. (2002) [30] demonstrated that the *MMP-1-1607 1G/2G* polymorphism in the *MMP-1* promoter region affects gene transcription and MMP-1 enzyme levels in non-malignant cells. This polymorphism has been linked to elevated levels of MMP-1 expression, which may have a detrimental effect on vascular integrity and tissue remodelling. Conversely, Okamoto et al. (2008) [31] demonstrated that transforming growth factor-beta 1 (TGF-β1) induces MMP-9 expression in meningeal cells. The present study highlighted the role of MMP-9 in ECM remodelling, a process which is crucial in the pathogenesis of IVH. Further studies, such as those by Schulz et al. (2004) [29], have investigated the activities of MMP-2 and MMP-9 in the plasma of preterm neonates. The findings suggest that there is elevated MMP-9 activity in critically ill preterm infants with BPD and/or IVH, indicating a potential involvement in the pathogenesis of these conditions. Additionally, studies conducted on a Polish cohort have demonstrated that polymorphisms in *MMP* genes are associated with ROP [16]. Moreover, research by Okamoto et al. (2010) [32] examined cerebrospinal fluid (CSF) levels of MMP-9 in infants with posthaemorrhagic hydrocephalus. The study revealed that patients with resolved ventricular dilation without shunt surgery exhibited significantly elevated CSF levels of MMP-9, suggesting a potential role for MMP-9 in the resolution of ventricular dilation following IVH.

In the context of the previously discussed roles of MMP-1 and MMP-9 in the pathogenesis of IVH in neonates, it is also important to consider their endogenous regulators, TIMP1 and TIMP2. These proteins are vital for regulating MMP activity, and any imbalance in their ratio may compromise vascular integrity and neural development, potentially contributing to IVH. The extant literature suggests a correlation between neonatal serum levels of TIMP2 and the risk of certain complications. For instance, Lee et al. (2015) [33] discovered that diminished TIMP2 concentrations were correlated with the subsequent progression of BPD. Although the present study did not specifically address IVH, it indicates that alterations in the MMP/TIMP system can affect neonatal tissue integrity. In a similar vein, Schulz et al. (2004) [29] demonstrated GA-dependent differences in plasma MMP and TIMP levels, reporting reduced TIMP1 concentrations in preterm infants compared to full-term neonates. These findings suggest that limited inhibitory capacity in premature neonates may increase the vulnerability of cerebral vessels to damage. Furthermore, Nikolov et al. (2020) [34] emphasised that TIMP1 and TIMP2 play a crucial role in regulating collagen turnover, placental remodelling, and vascular development. Dysregulation of this system has been linked to pregnancy complications, including impaired foetal growth and preeclampsia, further emphasising the importance of maintaining a balanced relationship between MMPs and TIMPs in ensuring vascular stability.

Several previous studies have investigated the influence of genetic variants on complications of prematurity. For example, a study of 342 preterm infants GA ≤ 28 weeks examined associations between vascular endothelial growth factor A (*VEGFA*) and nitric oxide synthase (*eNOS*) variants and the risk of IVH and ROP [35]. Similarly, the NICHD Neonatal Research Network Cytokines Study included 826 preterm infants to identify Single Nucleotide Polymorphisms (SNPs) associated with severe IVH [36]. Smaller cohorts have also been analysed; Kosik et al. studied 105 preterm infants GA < 32 weeks to explore vascular-related gene variants and their relationship with IVH [37], while a Polish cohort of 210 preterm infants GA < 33 weeks evaluated *ADRB2* polymorphisms in relation to ROP [38]. Collectively, these studies demonstrate a wide range of cohort sizes, from approximately 100 to over 800 infants, highlighting both the challenges and variability in power for detecting genetic associations in preterm populations.

The primary constraint of our study is the relatively modest sample size, which diminishes its capacity to discern statistically significant effects. Consequently, the majority of observed associations between *MMP-1*, *MMP-9*, *TIMP-1*, and *TIMP-2* variants and IVH failed to attain statistical significance. The T allele of *TIMP1 rs4898* was the only one to demonstrate a significant association with IVH. Given the exploratory nature of this study, these findings should be considered preliminary and require validation in larger, more diverse cohorts to confirm their statistical significance and ensure their clinical utility. Nevertheless, the findings of this study demonstrate that these variants do indeed exert an influence on the aforementioned condition. Moreover, the homogeneity of the study group, which consisted of Polish Caucasian neonates, serves to reinforce the significance of these findings. This study may provide an important contribution to future meta-analyses and highlights the need for further research in larger cohorts to clarify the role of *MMP/TIMP* polymorphisms in IVH.

## 4. Materials and Methods

### 4.1. Study Population

The current study utilised the same patient population previously characterised by Choręziak-Michalak et al. (2023) as well as the same methodological framework of this study [16]. A cohort of 100 preterm infants, entirely of Caucasian origin, was prospectively analysed at the Clinical Hospital of Gynaecology and Obstetrics in Poznan, from 1 March 2014 to 14 January 2020. Enrolment required both parental consent and GA between 22 + 0 and 33 + 0 weeks. The study population was divided into 48 IVH cases and 52 controls based on screening results. The severity of bleeding among the cases was then classified into four grades: Grade I (*n* = 1), Grade 2 (*n* = 21), Grade 3 (*n* = 22), and Grade 4 (*n* = 4).

Exclusion criteria for the study comprised infants (1) born from multiple pregnancies, (2) born from pregnancies involving the death of one of the foetuses, (3) with chromosomal abnormalities, (4) who reached death before 40 weeks of postmenstrual age and (5) diagnosed with inherited metabolic disorders.

### 4.2. Clinical Features

To identify potential drivers of IVH, we extracted various clinical parameters from the patients’ medical records. These were categorised into neonatal demographics (gender, GA in weeks, and BW in grams) and birth-related data, including pregnancy type (singleton vs. multiple), delivery mode, and Apgar scores at the 1st and 5th minutes. We also defined birth asphyxia specifically as an Apgar score below 6 at the 10th minute coupled with a cord blood pH < 7.0 or cord blood base excess (BE) < −15 mmol/L. Respiratory interventions were tracked by the type and duration of mechanical ventilation. The study also accounted for infectious and neonatal morbidities, ranging from intrauterine and late-onset infections (sepsis, urinary tract infections or pneumonia) to complications of prematurity such as BPD, ROP, and necrotizing enterocolitis (NEC).

### 4.3. Diagnostics

In Poland, transfontanelle ultrasound (TFUS) is routinely used as a screening tool for IVH in preterm infants. This method is highly sensitive and is typically performed in all neonates born before the 32nd week of gestation. The first ultrasound is conducted as soon as possible after birth to detect congenital brain abnormalities and haemorrhages. Follow-up examinations are routinely repeated throughout infancy to monitor potential changes. International guidelines support similar practices. In Poland, according to the Newborn Brain Society, first TFUS is being done directly after birth in order to identify congenital defects and haemorrhages. Next TFUS are repeated on the 3rd and 7th day of the life, then every week up to the 36th week of the adjusted age.

There is a classification of IVH among newborns, according to their extent- four-point Papilla scale [1,2,5]. Grade I is a haemorrhage limited to the germinal matrix, Grade II is IVH without ventricular dilatation, Grade III–IVH with ventricular dilatation occupying > 50% of the ventricle and Grade IV–IVH with intraparenchymal haemorrhage [1,2,5]. Grade II haemorrhages and their frequency are rarely documented and described. According to population studies their frequency is 5–19%. Haemorrhages more often described and documented are haemorrhages grade III and grade IV—their incidence in newborns born up to 28 weeks of pregnancy is 5–52% globally, 5–52% in Europe and 8–22% in North America [17]. Data from countries of the European Union inform that the frequency of III and IV grade IVH is 2–25%, on average 10% [11].

### 4.4. Treatment

Management of IVH in preterm infants primarily focuses on supportive care, as there is currently no specific causal treatment. This includes stabilisation of respiratory and cardiovascular functions, maintenance of optimal cerebral perfusion, and monitoring for signs of post-haemorrhagic ventricular dilation. In cases where hydrocephalus develops, surgical interventions such as ventricular reservoir placement or ventriculoperitoneal shunting may be necessary. Early neurodevelopmental follow-up is crucial, as infants with IVH are at increased risk for long-term neurological impairments. Multidisciplinary care and early intervention programmes can significantly improve developmental outcomes.

### 4.5. Data Collection

**Figure 1:**
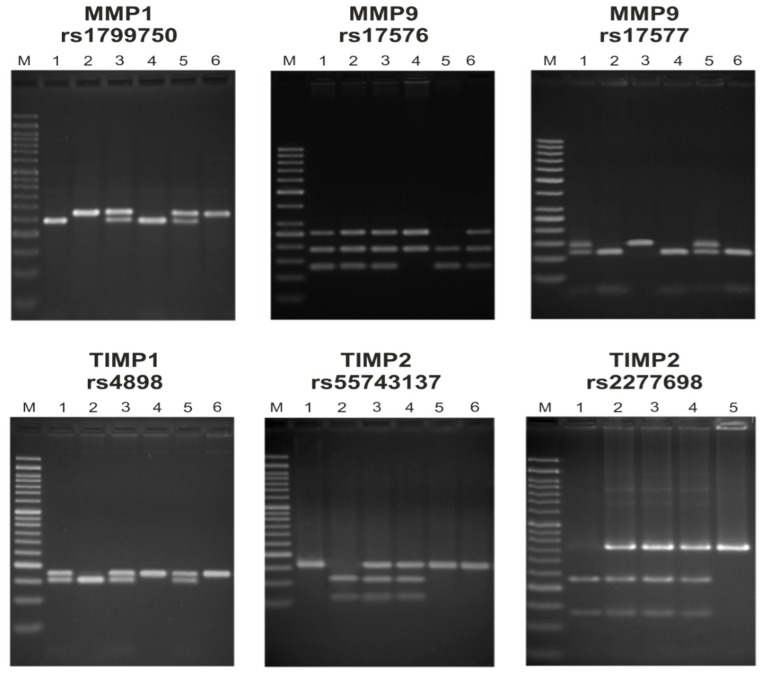
Examples RFLP reaction results: rs17577 MMP1 (lines 1, 4—1G/1G, 3, 5—1G/2G, 2, 6—2G/2G), rs17576 MMP9 (4—AA, 1, 2, 3, 6—AG, 5—GG), rs17577 MMP9 (2, 4, 6—GG, 2, 5—GA, 4—AA), rs4898 TIMP1 (4, 6—TT, 1, 3, 5—TC, 2—CC), rs2277698 TIMP2 (5—CC, 2, 3, 4—CT, 1—TT), rs55743137 TIMP2 (1, 5, 6—TT, 3, 4—TG, 2—GG), Lane M—50 bp marker used as molecular-weight reference in each gel. Lane numbers correspond to representative genotypes. The above RFLP analysis was performed for qualitative genotyping.

In our study we used data collected from peripheral venous blood samples taken after delivery and stored. Genomic DNA was extracted using the QIAamp DNA Blood Mini Kit (QIA-GEN Inc., Hilden, Germany) in accordance with manufacturer’s instructions. Polymerase chain reaction (PCR) and restriction fragment length polymorphism (RFLP) techniques were used to genotype the polymorphisms. Used primers and restriction enzymes are described in Table 6. Electrophoresis on agarose gels was performed using Midori Green Advance DNA Stain (Nippon Genetics, Düren, Germany)—Figure 1. For quality assurance, about 5% of the samples were blindly repeated. All variants showed a call rate exceeding 95%. 

## Figures and Tables

**Table 1:** Characteristics of patients.

Characteristics	I No-IVH (*n* = 52)	II IVH (*n* = 48)	II vs. I *p*-Value (Pearson)	IIa IVH Grade I (*n* = 1)	IIb IVH Grade II (*n* = 21)	IIc IVH Grade III (*n* = 22)	IId IVH Grade IV (*n* = 4)	IIa vs. IIb vs. IIc vs. IId *p*-Value (Pearson)
Sex, *n* (%)			*p* = 0.82230					*p* = 0.124669
Female	25 (48.07692)	22 (45.83333)		1 (4545)	6 (27.273)	12 (54.545)	3 (13.636)	
Male	27 (51.92308)	26 (54.16667)		0 (0)	15 (57.692)	10 (38.462)	1 (3846)	
Gestational age (weeks), median (range)	29 (24–32)	27 (22–33)	***p* = 0.000105**	28 (28–28)	29 (26–33)	25 (23–31)	23.5 (22–26)	***p* < 0.001**
Birth weight (grams), median (range)	1242.50 (550.000–1970.000)	955.000 (432.000–2010.000)	***p* = 0.002343**	1060.00 (1060.000–1060.000)	1230.000 (535.000–2010.000)	815.000 (432.000–1475.000)	710.000 (540.000–1000.000)	***p* = 0.000056**
Apgar score 1st minute, median (range)	6 (1–10)	4 (1–10)	***p* = 0.001859**	5 (5–5)	5 (1–10)	3 (1–8)	4.5 (1–6)	*p* = 0.2660
Apgar score 5th minute, median (range)	8 (5–10)	7 (1–10)	***p* = 0.003652**	8 (8–8)	8 (4–10)	7 (1–10)	6 (2–8)	***p* = 0.0181**
Mode of delivery, *n* (%)			*p* = 0.091219					*p* = 0.563498
Spontaneous Vaginal Delivery	15 (28.84615)	24 (50.00000)		0 (0)	8 (33.333)	13 (54.167)	3 (12.5)	
Caesarean section	36 (69.23077)	23 (47.91667)		1 (4.348)	12 (52.174)	9 (39.13)	1 (4.348)	
Vacuum	1 (1.92308)	1 (2.08333)		0 (0)	1 (100)	0 (0)	0 (0)	
Birth asphyxia, *n* (%)	2 (3.84615)	8 (16.66667)	***p* = 0.03432**	0 (0)	3 (37.5)	5 (62.5)	0 (0)	*p* = 0.644176
Mechanical ventilation, *n* (%)			***p* = 0.00031**					***p* = 0.00702**
non-invasive	35 (67.30769)	15 (31.25000)		0 (0)	12 (80)	2 (13.333)	1 (6.667)	
invasive	17 (32.69231)	33 (68.75000)		1 (3.03)	9 (27.273)	20 (60.606)	3 (9.091)	
Intrauterine infection/Early-onset infection, *n* (%)	23 (44.23077)	35 (72.91667)	***p* = 0.00369**	1 (2.857)	15 (42.857)	15 (42.857)	4 (11.429)	*p* = 0.545779
Late-onset infection, *n* (%)	6 (11.53846)	13 (27.08333)	*p* = 0.04774	0 (0)	5 (38.462)	7 (53.846)	1 (7.692)	*p* = 0.862823
ROP, *n* (%)	17 (11.53846)	33 (68.75000)	***p* = 0.00031**	1 (3.03)	10 (30.303)	19 (57.576)	3 (9.091)	***p* = 0.044614**
BPD, *n* (%)	11 (21.15385)	27 (56.25000)	***p* = 0.00030**	0 (0)	7 (25.926)	16 (59.259)	4 (14.815)	***p* = 0.010184**
NEC, *n* (%)	9 (17.30769)	12 (25.00000)	*p* = 0.34541	0 (0)	3 (25)	7 (58.333)	2 (16.667)	*p* = 0.321043

ROP—retinopathy of prematurity; BPD—bronchopulmonary dysplasia; NEC—necrotizing enterocolitis.

**Table 2:** Distribution of studied variants in IVH and no-IVH subjects with the analysis of differences in allele frequency, with multiple testing correction applied using the False Discovery Rate (FDR) according to the Holm–Bonferroni method.

Gene and Variant	I No-IVH		II IVH		Comparison of Groups II vs. I		Post Hoc Holm-Bonferroni
	*n*	%	*n*	%	OR (95% CI)	*p*-Value	
*MMP1 rs1799750*							
1G	46	44%	43	45%	1.023 (0.585–1.788)	0.936	1.000
2G	58	56%	53	55%	ref		
HWE *p*-value	0.112		0.829				
*MMP9 rs17576*							
A	64	62%	66	69%	ref		1.000
G	40	38%	30	31%	0.727 (0.405–1.606)	0.286	
HWE *p*-value	0.685		0.12				
*MMP9 rs17577*							
A	18	17%	13	14%	0.748 (0.345–1.624)	0.463	1.000
G	86	83%	83	86%	ref		
HWE *p*-value	0.589		0.883				
*TIMP1 rs4898*							
C	52	50%	33	34%	**0.524 (0.296–0.926)**	**0.026**	0.208
T	52	50%	63	66%	ref		
HWE *p*-value	0.579		0.667				
Female newborns							
C	24	48%	17	38%	0.681 (0.299–1.552)	0.362	1.000
T	26	52%	27	60%	ref		
HWE *p*-value	0.027		0.04				
Male newborns							
C	28	52%	16	31%	**0.413 (0.186–0.914)**	**0.029**	0.208
T	26	48%	36	69%	ref		
HWE *p*-value	0.004		0.157				
*TIMP2 rs2277698*							
C	95	91%	83	86%	ref		
T	9	9%	13	14%	1.653 (0.673–4.064)	0.273	1.000
HWE *p*-value	0.284		0.882				
*TIMP2 rs55743137*							
G	14	13%	16	17%	1.286 (0.591–2.799)	0.527	1.000
T	90	87%	80	83%	ref		
HWE *p*-value	0.945		0.488				

OR—Odds Ratio; CI—Confidence Interval; HWE—Hardy-Weinberg Equilibrium.

**Table 3:** Genotype distribution and analysis of the association between individual variants of *MMP-1*, *MMP-9*, *TIMP-1* and *TIMP-2* genes in IVH and no-IVH subjects, with multiple testing correction applied using the False Discovery Rate (FDR) according to the Holm–Bonferroni method.

Gene, SNP Variants and Tested Models	I No-IVH		II IVH		II vs. I in Codominant Model				Post Hoc Holm-Bonferroni
					Crude		Adjusted BW, GA, Mechanical Ventilation, ROP, APGAR 5′, BPD		
	*n*	%	*n*	%	OR (95% CI)	*p*-Value	AOR (95% CI)	*p*-Value	
*MMP1 rs1799750*									
1G/1G	13	25%	10	21%	0.974 (0.335–2.831)	0.962	0.747 (0.183–3.046)	0.684	1.000
1G/2G	20	38%	23	48%	1.457 (0.589–3.598)	0.415	1.708 (0.579–5.038)	0.333	1.000
2G/2G	19	37%	15	31%	ref				
Dominant	33	63%	33	69%	1.267 (0.496–3.235)	0.621	1.843 (0.610–5.563)	0.278	1.000
Recessive	13	25%	10	21%	0.789 (0.344–1.813)	0.577	0.737 (0.281–1.934)	0.535	1.000
Overdominant	32	62%	25	52%	0.679 (0.307–1.505)	0.341	0.493 (0.193–1.257)	0.138	0.690
*MMP9 rs17576*									
AA	19	37%	25	52%	ref				
AG	26	50%	16	33%	0.468 (0.197–1.108)	0.084	0.348 (0.121–1.000)	0.05	0.250
GG	7	13%	7	15%	0.76 (0.228–2.537)	0.656	0.531 (0.115–2.439)	0.415	1.000
Dominant	33	63%	23	48%	0.750 (0.225–2.496)	0.639	0.716 (0.157–3.269)	0.667	1.000
Recessive	7	13%	7	15%	0.857 (0.109–6.716)	0.883	0.295 (0.017–5.061)	0.4	1.000
Overdominant	26	50%	32	67%	1.273 (0.378–4.291)	0.697	0.967 (0.203–4.600)	0.966	1.000
*MMP9 rs17577*									
AA	1	2%	1	2%	0.972 (0.059–16.16)	0.984	6.544 (0.333–128.7)	0.217	1.000
GA	16	31%	11	23%	0.668 (0.272–1.640)	0.379	0.618 (0.215–1.772)	0.37	1.000
GG	35	67%	36	75%	ref				
Dominant	17	33%	12	25%	1.021 (0.277–3.765)	0.975	0.822 (0.164–4.115)	0.811	1.000
Recessive	1	2%	1	2%	3.52 × 10^−9^	0.998	4.28 × 10^−9^	0.998	1.000
Overdominant	36	69%	37	77%	0.762 (0.198–2.929)	0.692	0.907 (0.180–4.573)	0.906	1.000
*TIMP1 rs4898*									
Female newborns									
CC	3	12%	1	5%	0.222 (0.017–2.971)	0.256	0.425 (0.006–28.32)	0.689	1.000
TC	18	72%	15	68%	0.556 (0.132–2.342)	0.423	0.416 (0.063–2.762)	0.364	1.000
TT	4	16%	6	27%	ref				
Dominant	21	84%	16	73%	0.424 (0.056–3.212)	0.407	0.153 (0.002–9.624)	0.375	1.000
Recessive	3	12%	1	5%	3.94 × 10^−9^	0.998	1.27 × 10^−9^	0.999	1.000
Overdominant	7	28%	7	32%	0.964 (0.160–5.796)	0.968	0.668 (0.053–8.442)	0.755	1.000
Male newborns									
CC	11	41%	4	15%	0.260 (0.064–1.056)	0.06	0.305 (0.060–1.556)	0.153	0.508
TC	6	22%	8	31%	0.952 (0.251–3.615)	0.943	1.736 (0.262–11.51)	0.568	0.568
TT	10	37%	14	54%	ref				
Dominant	17	63%	12	46%	2.917 (0.407–20.90)	0.287	7.547 (0.264–215.6)	0.237	0.508
Recessive	11	41%	4	15%	0.208 (0.015–2.854)	0.24	0.046 (0.001–2.402)	0.127	0.508
Overdominant	21	78%	18	69%	1.09 × 10^−9^	0.998	-	-	
*TIMP2 rs2277698*									
CC	44	85%	36	75%	ref				
CT	7	13%	11	23%	1.921 (0.676–5.461)	0.221	2.134 (0.627–7.260)	0.225	1.000
TT	1	2%	1	2%	1.222 (0.074–20.23)	0.889	0.398 (0.018–8.693)	0.558	1.000
Dominant	8	15%	12	25%	1.833 (0.676–4.969)	0.234	1.750 (0.553–5.544)	0.341	1.000
Recessive	1	2%	1	2%	1.085 (0.066–17.85)	0.954	0.416 (0.020–8.690)	0.572	1.000
Overdominant	45	87%	37	77%	0.523(0.184–1.484)	0.223	0.471 (0.139–1.590)	0.225	1.000
*TIMP2 rs55743137*									
GG	1	2%	2	4%	2.294 (0.199–26.43)	0.506	1.723 (0.107–27.79)	0.701	1.000
TG	12	23%	12	25%	1.147 (0.456–2.887)	0.771	1.172 (0.398–3.445)	0.773	1.000
TT	39	75%	34	71%	ref				
Dominant	13	25%	14	29%	1.235 (0.510–2.990)	0.639	1.250 (0.454–3.443)	0.665	1.000
Recessive	1	2%	2	4%	2.217 (0.195–25.27)	0.521	1.937 (0.121–31.056)	0.641	1.000
Overdominant	40	77%	36	75%	0.9 (0.359–2.54)	0.822	0.865 (0.298–2.513)	0.79	1.000

OR—Odds Ratio; CI—Confidence Interval; AOR—Adjusted Odds Ratio; BW—birth weight; GA—gestational age; ROP—retinopathy of prematurity; BPD—bronchopulmonary dysplasia.

**Table 4:** Distribution of studied genes variants in patients IVH grade I + II and III + IV with frequency analysis of alleles.

Gene and Variant	I IVH Grade I + II		II IVH Grade III + IV		Comparison of Groups II vs. I	
	*n*	%	*n*	%	OR (95% CI)	*p*-Value
*MMP1 rs1799750*						
2G	21	48%	32	62%	ref	
1G	23	52%	20	38%	0.571 (0.253–1.288)	0.177
HWE *p*-value	0.992		0.898			
*MMP9 rs17576*						
A	32	73%	34	65%	ref	
G	12	27%	18	35%	1.412 (0.588–3.389)	0.44
HWE *p*-value	0.696		0.102			
*MMP9 rs17577*						
G	37	84%	46	88%	ref	
A	7	16%	6	12%	0.689 (0.213–2.229)	0.534
HWE *p*-value	0.48		0.506			
*TIMP1 rs4898*						
C	15	34%	18	35%	1.024 (0.439–2.384)	0.957
T	29	66%	34	65%	ref	
HWE *p*-value	0.674		0.334			
Female newborns						
C	6	43%	11	37%	0.772 (0.212–2.813)	0.695
T	8	57%	19	63%	ref	
HWE *p*-value	0.659		0.025			
Male newborns						
C	9	30%	7	32%	1.089 (0.332–3.577)	0.888
T	21	70%	15	68%	ref	
HWE *p*-value	0.424		0.218			
*TIMP2 rs2277698*						
C	38	86%	45	87%	ref	0.98
T	6	14%	7	13%	0.985 (0.305–3.183)	
HWE *p*-value	0.285		0.428			
*TIMP2 rs55743137*						
T	36	82%	44	85%	ref	
G	8	18%	8	15%	0.818 (0.279–2.396)	0.714
HWE *p*-value	0.07		0.354			

OR—Odds Ratio; CI—Confidence Interval.

**Table 5:** Genotype distribution and analysis of the association between individual variants of *MMP-1*, *MMP-9*, *TIMP-1* and *TIMP-2* genes and the grade of IVH of the studied patients.

Gene, SNP Variants and Tested Models	I IVH Grade I + II		II IVH Grade III + IV		II vs. I in Codominant Model			
					Crude		Adjusted BW, GA, Mechanical Ventilation, ROP, APGAR 5′, BPD	
	*n*	%	*n*	%	OR (95% CI)	*p*-Value	AOR (95% CI)	*p*-Value
*MMP1 rs1799750*								
1G/1G	6	27%	4	15%	0.333 (0.063–1.752)	0.194	2.67 × 10^−33^	0.999
1G/2G	11	50%	12	46%	0.545 (0.141–2.104)	0.379	1.515 (0.054–42.17)	0.807
2G/2G	5	23%	10	38%	ref		ref	
Dominant	17	77%	16	62%	2.063 (0.499–8.529)	0.312	13.709 (0.595–315.5)	0.102
Recessive	5	23%	10	38%	2.125 (0.595–7.584)	0.246	1.276 (0.197–8.275)	0.799
Overdominant	11	50%	14	54%	1.167 (0.374–3.637)	0.791	0.211 (0.008–5.367)	0.346
*MMP9 rs17576*								
AA	12	55%	13	50%	ref		ref	
AG	8	36%	8	31%	0.923 (0.263–3.239)	0.901	1.871 (0.129–26.93)	0.645
GG	2	9%	5	19%	2.308 (0.375–14.21)	0.367	6.042 (0.09–389.8)	0.398
Dominant	10	45%	13	50%	0.595 (0.114–3.102)	0.538	-	-
Recessive	2	9%	5	19%	2.83 × 10^8^	0.998	2.05 × 10^5^	0.999
Overdominant	14	64%	18	69%	3.6 (0.616–21.034)	0.155	-	-
*MMP9 rs17577*								
AA	1	5%		0%	0.268 (0.010–7.029)	0.43	0	0.998
GA	5	23%	6	23%	0.960 (0.247–3.728)	0.953	0.513 (0.037–7.122)	0.619
GG	16	73%	20	77%	ref		ref	
Dominant	6	27%	6	23%	0.583 (0.097–3.506)	0.555	0.045 (0–147.6)	0.452
Recessive	1	5%	0	0%	-	-	-	-
Overdominant	17	77%	20	77%	1.714 (0.285–10.30)	0.556	22.42 (0.007–74,197.7)	0.452
*TIMP1 rs4898*								
CC	3	14%	2	8%	0.667 (0.091–4.889)	0.69	1.34 × 10^8^	0.999
TC	9	41%	14	54%	1.556 (0.463–5.228)	0.475	4.124 (0.366–46.50)	0.252
TT	10	45%	10	38%	ref		ref	
Dominant	12	55%	16	62%	1.667 (0.308–9.014)	0.553	90.38 (0.01–879,207.5)	0.336
Recessive	3	14%	2	8%	9.47 × 10^7^	0.997	1.70 × 10^5^	0.998
Overdominant	13	59%	12	46%	0.857(0.165–4.477)	0.855	0.012 (0–96.02)	0.334
Female newborns								
CC	1	14%		0%	2.24 × 10^−9^	0.998	-	-
TC	4	57%	11	73%	1.375 (0.178–10.65)	0.76	0.276 (0–4,082,234)	0.879
TT	2	29%	4	27%	ref			
Dominant	5	71%	11	73%	5 (0.273–91.52)	0.278	3.98 × 10^10^	0.999
Recessive	1	14%	0	0%	-	-	-	-
Overdominant	3	43%	4	27%	0.2 (0.01–3.66)	0.278	2.51 × 10^−11^	0.999
Male newborns								
CC	2	13%	2	18%	1.333 (0.144–12.36)	0.8	-	-
TC	5	33%	3	27%	0.8 (0.135–4.745)	0.806	-	-
TT	8	53%	6	55%	ref			
Dominant	7	47%	5	45%	0.75 (0.084–6.71)	0.797	5.49 × 10^6^	0.999
Recessive	2	13%	2	18%	3.27 × 10^8^	0.998	0.014	0.999
Overdominant	10	67%	8	73%	2.667 (0.77–25.64)	0.396	0	0.999
*TIMP2 rs2277698*								
CC	17	77%	19	73%	ref		ref	
CT	4	18%	7	27%	1.566 (0.389–6.298)	0.528	1.462 (0.066–32.54)	0.81
TT	1	5%		0%	0.299 (0.011–7.832)	0.469	0.00 × 10^0^	0.998
Dominant	5	23%	7	27%	1.253 (0.334–4.694)	0.74	1.014 (0.064–15,98)	0.992
Recessive	1	5%	0	0%	3.47 × 10^−9^	0.998	0.00 × 10^0^	0.998
Overdominant	18	82%	19	73%	0.603 (0.151–2.415)	0.475	0.716 (0.03–16.56)	0.835
*TIMP2 rs55743137*								
GG	2	9%		0%	0.178 (0.008–3.992)	0.277	0	0.998
TG	4	18%	8	31%	1.778 (0.449–7.040)	0.413	0.872 (0.072–10.55)	0.915
TT	16	73%	18	69%	ref		ref	
Dominant	6	27%	8	31%	1.185 (0.338–4.156)	0.791	0.739 (0.067–8.157)	0.805
Recessive	2	9%	0	0%	3.31 × 10^−9^	0.998	0	0.998
Overdominant	18	82%	18	69%	0.500 (0.128–1.961)	0.32	1.132 (0.091–14.105)	0.924

OR—Odds Ratio; CI—Confidence Interval; AOR—Adjusted Odds Ratio; BW—birth weight; GA—gestational age; ROP—retinopathy of prematurity; BPD—bronchopulmonary dysplasia.

**Table 6:** Primers and PCR-RFLP conditions for studied genetic variants.

Gene and Variant	Sequence of Primers	Temperature of Primer Attachment	Restriction Enzyme	PCR Products
MMP-1 rs179975	5′-TGACTTTTAAAACATAGTCTATGTTCA-3′ 5′-TCTTGGATTGATTTGAGATAAGTCATAGC-3′	50 °C	AluI	1G 241, 28 bp 2G 269 bp
MMP-9 rs17576	5′-GAGAGATGGGATGAACTG-3′ 5′-GTGGTGGAAATGTGGTGT-3′	60 °C	MspI (HpaII)	A 252, 187 bp G 187, 129, 123 bp
MMP-9 rs17577	5′-ACACGCACGACGTCTTCCAGTATC-3′ 5′-GGGGCATTTGTTTCCATTTCCA-3′	63 °C	TaqI	G 115, 23 bp A 138 bp
TIMP-1 rs4898	5′-GCACATCACTACCTGCAGTCT-3′ 5′-GAAACAAGCCCACGATTTAG-3′	54 °C	BauI (BssI)	T 175 bp C 153, 22 bp
TIMP-2 rs2277698	5′-CCAGGAAATTGGCAGGTAGT-3′ 5′-GAATTCACCAACTGTGTGGC-3′	60 °C	BsrI	C 369 bp T 231, 138 bp
TIMP-2 rs55743137	5′-CCTTTGAACATCTGGAAAGACAA-3′ 5′-TAACCCATGTATTTGCACTTCCT-3′	58 °C	AluI	T 160 bp G 108, 52 bp

## Data Availability

The original contributions presented in this study are included in the article/Supplementary Materials. Further inquiries can be directed to the corresponding author.

## Supplementary Materials

The following supporting information can be downloaded at: https://www.mdpi.com/article/10.3390/ijms27062596/s1.

## Abbreviations

The following abbreviations are used in this manuscript:

AOR	Adjusted odds ratio
BBB	Blood–brain barrier
BE	Blood base excess
BPD	Bronchopulmonary dysplasia
BW	Birth weight
CSF	Cerebrospinal fluid
ECM	Extracellular matrix
eNOS	Nitric oxide synthase
FDR	False Discovery Rate
GA	Gestational age
HWE	Hardy–Weinberg equilibrium
IVH	Intraventricular haemorrhage
MMPs	Metalloproteinases
NEC	Necrotizing enterocolitis
OR	Odds ratio
PCR	Polymerase chain reaction
RFLP	Restriction fragment length polymorphism
ROP	Retinopathy of prematurity
SNPs	Single Nucleotide Polymorphisms
TFUS	Transfontanelle ultrasound
TGF-β1	Transforming growth factor-beta 1
TIMPs	Tissue inhibitors of metalloproteinases
VEGFA	Vascular endothelial growth factor A

## References

[1] Bokiniec R., Szczapa J. (2008). Podstawy Neonatologii.

[2] Starr R., De Jesus O., Shah S.D., Borger J. (2025). Periventricular and Intraventricular Hemorrhage. StatPearls.

[3] Hand I.L., Shellhaas R.A., Milla S.S., Cummings J.J., Adams-Chapman I.S., Aucott S.W., Goldsmith J.P., Kaufman D.A., Martin C.R., Committee on Fetus and Newborn, Section on Neurology, Section on Radiology (2020). Routine Neuroimaging of the Preterm Brain. Pediatrics.

[4] Pediatria Po Dyplomie-Noworodek Urodzony Przedwcześnie z Uszkodzeniem Mózgu. https://podyplomie.pl/pediatria/12184,noworodek-urodzony-przedwczesnie-z-uszkodzeniem-mozgu-problemy-okresu-niemowlecego-i-wczesnego?srsltid=AfmBOoq3Km8bgjhiKcG53Qd3IkuXmKyqbUiyQ211O4bfyybpamOtSmB.

[5] Intraventricular Hemorrhage in the Preterm Infant: Background, Pathophysiology, Etiology. https://emedicine.medscape.com/article/976654-overview?form=fpf.

[6] Szpecht D., Wiak K., Braszak A., Szymankiewicz M., Gadzinowski J. (2016). Role of Selected Cytokines in the Etiopathogenesis of Intraventricular Hemorrhage in Preterm Newborns. Childs Nerv. Syst. ChNS Off. J. Int. Soc. Pediatr. Neurosurg..

[7] Tsao P.-C. (2023). Pathogenesis and Prevention of Intraventricular Hemorrhage in Preterm Infants. J. Korean Neurosurg. Soc..

[8] El-Atawi K. (2016). Risk Factors, Diagnosis, and Current Practices in the Management of Intraventricular Hemorrhage in Preterm Infants: A Review. Acad. J. Pediatr. Neonatol..

[9] Lee J.Y., Kim H.S., Jung E., Kim E.S., Shim G.H., Lee H.J., Lee J.A., Choi C.W., Kim E.-K., Kim B.I. (2010). Risk Factors for Periventricular-Intraventricular Hemorrhage in Premature Infants. J. Korean Med. Sci..

[10] Kosik K., Szpecht D., Al-Saad S.R., Karbowski L.M., Kurzawińska G., Szymankiewicz M., Drews K., Wolski H., Seremak-Mrozikiewicz A. (2020). Single Nucleotide Vitamin D Receptor Polymorphisms (FokI, BsmI, ApaI, and TaqI) in the Pathogenesis of Prematurity Complications. Sci. Rep..

[11] Zimbeck M., Mohangoo A., Zeitlin J. (2009). EURO-PERISTAT Report Writing Committee The European Perinatal Health Report: Delivering Comparable Data for Examining Differences in Maternal and Infant Health. Eur. J. Obstet. Gynecol. Reprod. Biol..

[12] Hadler-Olsen E., Fadnes B., Sylte I., Uhlin-Hansen L., Winberg J.-O. (2011). Regulation of Matrix Metalloproteinase Activity in Health and Disease. FEBS J..

[13] Shapiro S.D., Senior R.M. (1999). Matrix Metalloproteinases. Matrix Degradation and More. Am. J. Respir. Cell Mol. Biol..

[14] Dragun P., Makarewicz D., Wójcik L., Ziemka-Nałecz M., Słomka M., Zalewska T. (2008). Matrix Metaloproteinases Activity during the Evolution of Hypoxic-Ischemic Brain Damage in the Immature Rat. The Effect of 1-Methylnicotinamide (MNA). J. Physiol. Pharmacol. Off. J. Pol. Physiol. Soc..

[15] Gilard V., Tebani A., Bekri S., Marret S. (2020). Intraventricular Hemorrhage in Very Preterm Infants: A Comprehensive Review. J. Clin. Med..

[16] Choręziak-Michalak A., Szpecht D., Chmielarz-Czarnocińska A., Seremak-Mrozikiewicz A., Drews K., Kurzawińska G., Strauss E., Gotz-Więckowska A. (2023). Comprehensive Analysis of the Role of Gene Variants in Matrix Metalloproteinases and Their Tissue Inhibitors in Retinopathy of Prematurity: A Study in the Polish Population. Int. J. Mol. Sci..

[17] Siffel C., Kistler K.D., Sarda S.P. (2021). Global Incidence of Intraventricular Hemorrhage among Extremely Preterm Infants: A Systematic Literature Review. J. Perinat. Med..

[18] Preterm Birth. https://www.who.int/news-room/fact-sheets/detail/preterm-birth.

[19] Zhu Y., Spitz M.R., Lei L., Mills G.B., Wu X. (2001). A Single Nucleotide Polymorphism in the Matrix Metalloproteinase-1 Promoter Enhances Lung Cancer Susceptibility. Cancer Res..

[20] Hu Z., Huo X., Lu D., Qian J., Zhou J., Chen Y., Xu L., Ma H., Zhu J., Wei Q. (2005). Functional Polymorphisms of Matrix Metalloproteinase-9 Are Associated with Risk of Occurrence and Metastasis of Lung Cancer. Clin. Cancer Res. Off. J. Am. Assoc. Cancer Res..

[21] Wolosowicz M., Prokopiuk S., Kaminski T.W. (2025). Matrix Metalloproteinase-9 (MMP-9) as a Therapeutic Target: Insights into Molecular Pathways and Clinical Applications. Pharmaceutics.

[22] Lorente L., Martín M., Plasencia F., Solé-Violán J., Blanquer J., Labarta L., Díaz C., Borreguero-León J.M., Jiménez A., Páramo J.A. (2013). The 372 T/C Genetic Polymorphism of TIMP-1 Is Associated with Serum Levels of TIMP-1 and Survival in Patients with Severe Sepsis. Crit. Care.

[23] Lee C.-I., Lee Y.-J., Lee T.-H., Lee C.-Y., Tsao H.-M., Cheng E.-H., Huang C.-C., Yang S.-F., Lee M.-S. (2025). TIMP2 Rs2277698 Polymorphism Associated with Adverse IVF Outcomes in Han Chinese Women. Front. Endocrinol..

[24] Rs55743137 RefSNP Report-dbSNP-NCBI. https://www.ncbi.nlm.nih.gov/snp/rs55743137#publications.

[25] Helwich E., Rutkowska M., Bokiniec R., Gulczyńska E., Hożejowski R. (2017). Intraventricular Hemorrhage in Premature Infants with Respiratory Distress Syndrome Treated with Surfactant: Incidence and Risk Factors in the Prospective Cohort Study. Dev. Period Med..

[26] Kosik K., Sowińska A., Seremak-Mrozikiewicz A., Abu-Amara J.A., Al-Saad S.R., Karbowski L.M., Gryczka K., Kurzawińska G., Szymankiewicz-Bręborowicz M., Drews K. (2022). Polymorphisms of Fibronectin-1 (Rs3796123; Rs1968510; Rs10202709; Rs6725958; and Rs35343655) Are Not Associated with Bronchopulmonary Dysplasia in Preterm Infants. Mol. Cell. Biochem..

[27] Cockle J.V., Gopichandran N., Walker J.J., Levene M.I., Orsi N.M. (2007). Matrix Metalloproteinases and Their Tissue Inhibitors in Preterm Perinatal Complications. Reprod. Sci. Thousand Oaks Calif.

[28] Lattanzi S., Di Napoli M., Ricci S., Divani A.A. (2020). Matrix Metalloproteinases in Acute Intracerebral Hemorrhage. Neurotherapeutics.

[29] Schulz C.G., Sawicki G., Lemke R.P., Roeten B.M., Schulz R., Cheung P.-Y. (2004). MMP-2 and MMP-9 and Their Tissue Inhibitors in the Plasma of Preterm and Term Neonates. Pediatr. Res..

[30] Fujimoto T., Parry S., Urbanek M., Sammel M., Macones G., Kuivaniemi H., Romero R., Strauss J.F. (2002). A Single Nucleotide Polymorphism in the Matrix Metalloproteinase-1 (MMP-1) Promoter Influences Amnion Cell MMP-1 Expression and Risk for Preterm Premature Rupture of the Fetal Membranes. J. Biol. Chem..

[31] Okamoto T., Takahashi S., Nakamura E., Nagaya K., Hayashi T., Shirai M., Fujieda K. (2008). Matrix Metalloproteinases in Infants with Posthemorrhagic Hydrocephalus. Early Hum. Dev..

[32] Okamoto T., Takahashi S., Nakamura E., Nagaya K., Hayashi T., Shirai M., Fujieda K. (2010). Increased Expression of Matrix Metalloproteinase-9 and Hepatocyte Growth Factor in the Cerebrospinal Fluid of Infants with Posthemorrhagic Hydrocephalus. Early Hum. Dev..

[33] Lee C., An J., Kim J.H., Kim E.S., Kim S.H., Cho Y.K., Cha D.H., Han M.Y., Lee K.H., Sheen Y.H. (2015). Low Levels of Tissue Inhibitor of Metalloproteinase-2 at Birth May Be Associated with Subsequent Development of Bronchopulmonary Dysplasia in Preterm Infants. Korean J. Pediatr..

[34] Nikolov A., Popovski N., Hristova I. (2020). Collagenases MMP-1, MMP-13, and Tissue Inhibitors TIMP-1, TIMP-2: Their Role in Healthy and Complicated Pregnancy and Potential as Preeclampsia Biomarkers—A Brief Review. Appl. Sci..

[35] Poggi C., Giusti B., Gozzini E., Sereni A., Romagnuolo I., Kura A., Pasquini E., Abbate R., Dani C. (2015). Genetic Contributions to the Development of Complications in Preterm Newborns. PLoS ONE.

[36] Thornburg C.D., Erickson S.W., Page G.P., Clark E.A., DeAngelis M.M., Hartnett M.E., Goldstein R.F., Dagle J.M., Murray J.C., Poindexter B.B. (2021). Genetic Predictors of Severe Intraventricular Hemorrhage in Extremely Low-Birthweight Infants. J. Perinatol..

[37] Kosik K., Szpecht D., Karbowski Ł., Al-Saad S.R., Chmielarz-Czarnocińska A., Minta M., Sowińska A., Strauss E. (2023). Hemangioma-Related Gene Polymorphisms in the Pathogenesis of Intraventricular Hemorrhage in Preterm Infants. Childs Nerv. Syst..

[38] Chmielarz-Czarnocińska A., Durska A., Skulimowski B., Sobaniec A., Gotz-Więckowska A., Strauss E. (2025). Association of the ADRB2 Rs1042714 Variant with Retinopathy of Prematurity Highlights the Importance of the Renin-Angiotensin-Aldosterone System. Sci. Rep..

