# Supplementary material for: Association of Gene Variants in Matrix Metalloproteinases and Their Tissue Inhibitors with Intraventricular Haemorrhage in Preterm Infants
Source: Int J Mol Sci. 2026 Mar 12;27(6):2596. doi: 10.3390/ijms27062596 (PMC13026099; doi:10.3390/ijms27062596)
Supplement: Supplementary file 1 [file ijms-27-02596-s001.zip › ijms-4100452-supplementary 25.02.pdf]

An additional correction for multiple testing using the False Discovery Rate (FDR) was applied to post hoc pairwise comparisons performed only for results that were statistically significant in the primary analysis. Due to the small sample size, infants with IVH grade I (n = 1) were excluded from the comparative analyses across IVH severity grades.

For GA and BW, statistically significant differences between IVH grade 2 and grades 3–4 remained significant after FDR correction (Table S1., Table S2.). For the 5 minute Apgar score, the difference between IVH grade 2 and grade 3 remained statistically significant, while no additional pairwise differences reached significance (Table S3.). For mechanical ventilation, the association between IVH grades 2 and 3 remained statistically significant (p=0,0046). Infants requiring invasive ventilation were more likely to have IVH grade 3 compared with non-invasive ventilation, whereas infants receiving non-invasive ventilation were more frequently classified as IVH grade 2compared with invasive (Table S4.). Similarly, for ROP, the difference between IVH grades 2 and 3 remained statistically significant (p=0,04). Infants who developed ROP were more likely to have IVH grade 3 that IVH grade 2 (Table S5.). For BPD, no pairwise comparisons reached statistical significance after FDR correction. Although, the association between IVH grades 2 and 3 approached significance (p = 0,058), suggesting a trend toward higher IVH grades in infants who developed BPD (Table S6.).

Table S1. post hoc Dunn-Bonferroni

Gestational age

**p-value**	**IVH grade**		
	**II**	**III**	**IV**
**II**		**0,000012**	**0,000565**
**III**	**0,000012**		0,740084
**IV**	**0,000565**	0,740084	

Table S2. post hoc Tukey HSD

Birth weight

**p-value**	**IVH grade**		
	**II**	**III**	**IV**
**II**		**0,000143**	**0,00717**
**III**	**0,000143**		0,791129
**IV**	**0,00717**	0,791129	

Table S3. post hoc Dunn-Bonferroni

5 minute Apgar score

p-value	IVH grade		
	**II**	**III**	**IV**
**II**		**0,029024**	0,195815
**III**	**0,029024**		1
**IV**	0,195815	1	

Table S4. post hoc Bonferroni-Holm

Mechanical ventilation

p-value	IVH grade		
	**II**	**III**	**IV**
**non-invasive**	80%	13,333%	6,667%
**invasive**	27,273%	60,606%	9,091%

Table S5. post hoc Bonferroni-Holm

ROP

p-value	IVH grade		
	**II**	**III**	**IV**
**yes**	30,303%	57,576%	9,091%
**no**	73,333%	20%	6,667%

Table S6. post hoc Bonferroni-Holm

BPD

**p-value**	**IVH grade**		
	**II**	**III**	**IV**
**yes**	25,259%	59,259%	14,815%
**no**	66,667%	28,571%	4,762%
